# Defining Duplex Ultrasound Criteria for In-Stent Restenosis of the Carotid Artery Using Computed Tomographic Angiography

**DOI:** 10.7759/cureus.26700

**Published:** 2022-07-09

**Authors:** Lucas J Bitsko, Evan J Ryer, Ellen P Penn, Gregory G Salzler, Matthew Major, Jeremy Irvan, James R Elmore

**Affiliations:** 1 Endovascular and Vascular Surgery, Geisinger Medical Center, Danville, USA

**Keywords:** duplex ultrasound, ct angiogram, restenosis, carotid artery stenting, carotid in-stent restenosis

## Abstract

Introduction

Duplex ultrasound (DUS) velocity measurement is the preferred method for evaluating carotid artery stenosis. However, velocity criteria based upon native carotid arteries may not apply to internal carotid artery stents. Previously, catheter-based angiography was used to determine DUS velocity criteria for in-stent restenosis (ISR), but conventional angiography is invasive and can be limited. This study sought to define duplex ultrasound velocity criteria for predicting internal carotid artery in-stent restenosis by correlating in-stent velocities with computed tomographic angiography (CTA) measurements of percent stenosis.

Methods

A retrospective chart review was conducted on all patients who underwent internal carotid artery (ICA) stenting within our health system between January 2013 and February 2020. Thirty-eight surveillance DUS studies from 32 patients were found to have CTA performed within 30 days. Centerline reconstructions of internal carotid artery stents were created using Aquarius iNtuition software (TeraRecon, Durham, NC, USA). Two independent observers measured percent stenosis by three built-in methods. Stenotic areas were matched to DUS-measured peak systolic velocities (PSV) and end-diastolic velocities (EDV). Internal carotid artery PSV (stent) to common carotid artery (CCA) PSV ratios (ICA/CCA) were calculated, and receiver operating characteristic (ROC) curves were generated. The optimal DUS velocity criteria in the stented ICA were determined by maximizing Youden’s index.

Results

Mean vessel diameter measurement of percent stenosis resulted in the most accurate model for all DUS velocity parameters (PSV, EDV, and ICA/CCA ratio) and was used for threshold determinations (area under the receiver operating characteristics (AUROC): 0.99, 0.96, and 0.96, respectively). A PSV cutoff of 240 cm/s for ≥60% ISR resulted in the highest Youden’s index (97%) with 100% sensitivity and 97% specificity. Secondary DUS parameters included an EDV ≥50 cm/s (Youden’s index 84%) and an ICA/CCA ratio ≥ 2.2 (Youden’s index 91%).

Conclusions

Velocity criteria to predict internal carotid artery ISR is needed to inform decisions for possible reintervention. Using CTA, we found that a PSV ≥240 cm/s on carotid DUS can predict ≥60% ISR with high sensitivity and specificity. This value can be used as an alternative to current velocity criteria based on native carotid arteries. However, the optimal thresholds for EDV and ICA/CCA ratio were similar to native carotid arteries.

## Introduction

Transfemoral carotid artery stenting (CAS) is an effective treatment for carotid artery disease, albeit with a higher periprocedural stroke rate when compared with carotid endarterectomy (CEA) [1-4]. Recently, there has been a resurgence in carotid stenting due to transcarotid artery revascularization (TCAR) with flow-reversal, which has demonstrated improved short-term outcomes [5]. Moreover, CAS has always compared favorably to CEA with regard to long-term outcomes [6,7], which suggests CAS will continue to be an option for the treatment of carotid artery disease.

Important in the follow-up of carotid stent patients is the assessment of in-stent restenosis (ISR). In the evaluation of stenosis in unstented carotid arteries, traditional digital subtraction angiography (DSA) has largely been replaced by other modalities, such as computed tomography angiography (CTA), magnetic resonance angiography (MRA), and duplex ultrasound (DUS) [8-10]. With regards to DUS, the parameters for evaluation of carotid artery disease are peak systolic velocity (PSV), end-diastolic velocity (EDV), and the ratio between peak systolic velocities in the internal carotid artery (ICA) and common carotid artery (CCA) [11]. However, the use of validated criteria for native carotid arteries may overestimate the degree of ISR, as it has been suggested that stent placement alters flow characteristics and increases baseline velocity for given percent stenosis [12-14].

Previous work has been done to refine carotid artery DUS velocity criteria for ISR predominately using catheter-based angiography [15-17], but DSA is limited by single-plane projection. Computed tomography angiography offers the ability to obtain 3D reconstructions and centerline measurements for vessel lumen segmentation, and is accurate in the evaluation of carotid stenosis [18,19]. Other studies have used CTA in addition to DSA [20] or were unable to draw conclusions about severe stenosis [21]. We hypothesize that carotid DUS velocity criteria can be better defined for internal carotid artery ISR by correlating in-stent velocities with percent stenosis using CTA centerline reconstructions.

This article was previously presented as a meeting abstract at the 35th Annual Meeting of the Eastern Vascular Society on September 25th, 2021.

## Materials and methods

General methodology

This work was conducted as a retrospective single-center cohort study: a retrospective chart review was conducted on all patients (406) who underwent internal carotid artery (ICA) stenting within our health system between January 2013 and February 2020. Approval for this study was granted after review by the Geisinger Institutional Review Board (reference number 2020-0119). Informed consent was not required. Patients were identified from the electronic medical record (EMR) using the International Classification of Diseases - Ninth Revision (ICD-9) and current procedural terminology (CPT) codes. Demographics, clinical characteristics, radiologic, and operative data were obtained from the EMR. Patients who had postoperative CTA and DUS performed within 30 days were included in the study (i.e., paired studies). We excluded patients who received an ipsilateral common carotid artery stent, had a contralateral carotid occlusion, had a poor-quality duplex ultrasound, or those with severe artifact on CTA. For statistical analysis, each paired study was considered a separate sample.

Duplex ultrasound

All carotid DUS were obtained in Intersocietal Accreditation Commission (IAC) accredited vascular laboratories on Logiq E9 (GE Healthcare, Chicago, USA) ultrasound machines. Grayscale and color images of the carotid arteries were obtained. Peak systolic and end-diastolic velocities were obtained at the proximal, mid, and distal ICA stent, along with areas of visible stenosis. Common carotid artery (CCA) peak systolic velocities were obtained 2 cm proximal to the carotid bifurcation. All DUS studies were reviewed by two independent, fellowship-trained vascular surgeons who were blinded to patient identity.

Computed tomographic angiography

**Figure 1:**
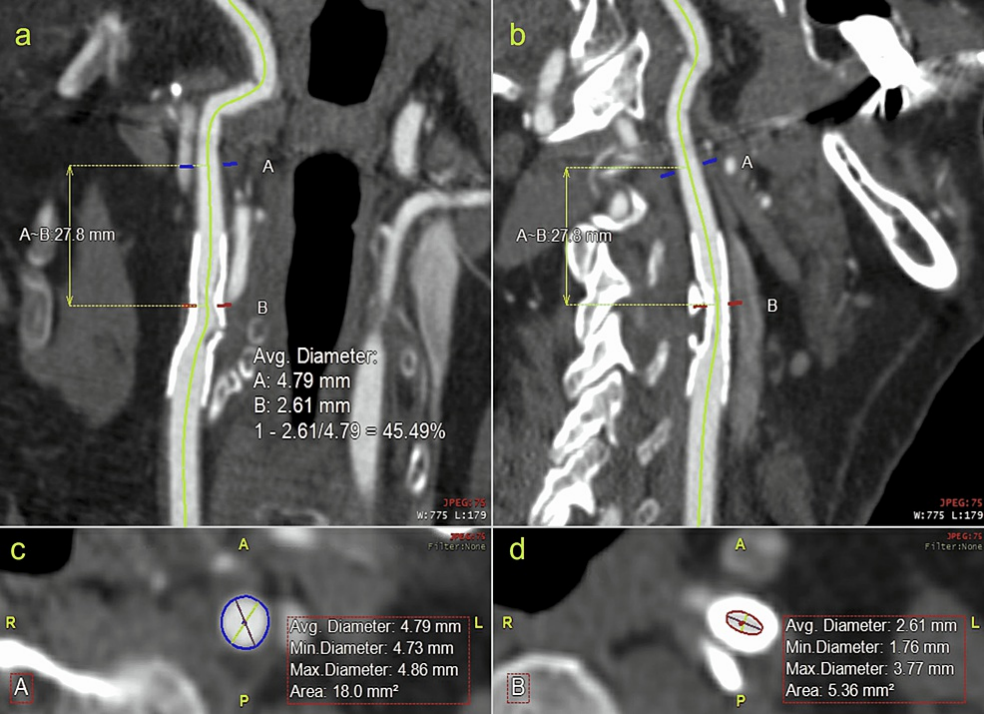
Centerline reconstruction of stented internal carotid artery Centerline measurements of the ICA from CTA in (a) sagittal, and (b) coronal projection with the most stenotic portion noted at the red hash compared with normal artery diameter noted by the blue hash. Axial illustration of the stent with flow channels selected at the (c) normal distal portion of the ICA, and (d) the most stenotic in-stent portion.

Multidetector-row computed tomographic angiogram (MDCTA) of the neck at a slice thickness of 1.25 mm or less was used to visualize the entire stented ICA. Advanced 3D reconstructions of carotid and branch vessels were performed for optimal vascular evaluation on a separate workstation. Metal artifact reduction algorithms were applied in all studies. Centerline reconstructions were obtained using the Aquarius iNtuition software (TeraRecon Inc., Durham, NC, USA) (Figure 1). Software-aided identification of the vessel lumen was performed by threshold segmentation refined by two independent, fellowship-trained vascular surgeons who were blinded to the duplex report. The surgeons identified the most stenotic area within the stent and a portion of the distal ICA with normal flow diameter as per the North American Symptomatic Carotid Endarterectomy Trial (NASCET) protocol [22].

Determining percent stenosis and corresponding DUS parameters

Percent stenosis was computed by the Aquarius iNtuition software using three built-in measurement methods: mean vessel diameter (MVD) i.e., the average of the major and minor axis at stenosis divided by diameter in normal artery distal to the stent; minimum vs maximum diameter (MvM) i.e., the minimum axis diameter at stenosis divided by maximum axis diameter in normal artery distal to stent); and percent area reduction (PAR) i.e., the area at stenosis divided by area in normal artery distal to the stent. The percent stenosis used for statistical analysis was the average of measurements taken by two independent vascular surgeons. In the event the two measurements differed by ≥20%, the measurement would have been repeated by a third, blinded vascular surgeon, but this never occurred. In-stent restenosis was categorized by the percent decrease in flow lumen <60% and ≥60%. Peak systolic and end-diastolic velocities from internal carotid DUS were matched to the location of maximum ISR on CTA. The ICA/CCA ratios were calculated by dividing the in-stent PSV by the peak systolic velocity in the ipsilateral CCA.

Statistics

Statistical analysis was conducted using SAS® Enterprise Guide 8.2: User’s Guide (SAS Institute Inc., Cary, NC, USA). Interobserver reliability for each CTA measurement technique was determined by the intraclass correlation coefficient (ICC). Student’s t-tests were performed to compare PSV means for <60% and ≥60% ISR cohorts identified by CTA and to compare means for quantitative demographic data. Chi-square tests of independence were used to compare frequency distributions for categorical demographic data. Multiple one-way analysis of variances (ANOVAs) were used to independently compare means between known stent types for each CTA measurement method (MVD, MvM diameter, and PAR) and DUS velocity parameter (PSV, EDV, and ICA/CCA ratio). Receiver operating characteristic (ROC) curves were generated and the area under the ROC (AUROC) curve was calculated for each ISR measurement technique and DUS parameter. Within each ISR measurement group, AUROC differences between PSV, EDV, and ICA/CCA ratios were analyzed by the method of DeLong et al. [23]. Correction for multiple comparisons was performed by controlling the false discovery rate [24]. Using MVD as our standard for percent stenosis, sensitivity, specificity, positive predictive value (PPV), negative predictive value (NPV), and Youden's index were calculated for a variety of PSV, EDV, and ICA/CCA ratio cutoff values. Optimal cutoffs to predict a ≥60% ISR were determined by the maximum Youden’s index. Combination criteria were not assessed. A P-value of <0.05 was considered statistically significant.

## Results

Patient characteristics

Four hundred and six patients were identified with carotid stents over the study period. From this population, 32 patients with 38 paired studies were identified. The demographics of this subset were generally similar to all CAS patients identified (Table 1). A greater percentage of males were present in the subset population than the total population (81% vs 66%, p=0.02). Computed tomography angiography (CTA) was performed at an average of 472 days post-stenting in the <60% ISR cohort and at an average of 604 days for the ≥60% ISR cohort. Indications for CTA varied, with 28 studies (74%) being performed due to vascular disease present outside the stented vessel, eight (21%) due to elevated DUS velocities within the stent, one (3%) due to head/neck carcinoma, and one (3%) in the setting of trauma.

**Table 1:** Demographic data of patients comparing the study population to the sample population DUS: Duplex ultrasound, CTA: Computed tomographic angiography

	Study Population	30-day paired DUS & CTA	P-value
Total Patients	406	32	
Male	262 (66%)	30 (81%)	0.02
Female	144 (34%)	7 (19%)	
White Non-Hispanic	399 (98%)	32 (100%)	0.99
Average age at procedure (years)	69 ± 9.09 (25-92)	69 ± 9.51 (39-84)	0.86
Comorbidities			
Coronary Artery Disease	202 (50%)	21 (66%)	0.08
Diabetes Mellitus	164 (40%)	7 (19%)	0.03
Hypertension	342 (84%)	29 (91%)	0.48
Smoking History	331 (82%)	26 (81%)	0.96
Chronic Kidney Disease	90 (22%)	2 (6%)	0.06

At the time of the procedure, the average age in the subset population was 69 ± 9.51 versus 69 ± 9.09 (p=0.86). Similar percentages of the subset and total population had hypertension (91% vs 84 %, p=0.48), coronary artery disease (66% vs 50%, p=0.08), chronic kidney disease (6% vs 22%, p=0.06), and a history of tobacco use (81% vs 82%, p= 0.96). Fewer patients in the subset cohort had diabetes mellitus (19% vs 40%, p=0.03). No patients in the sample population had peri-procedural strokes or had reintervention performed after surveillance imaging.

Stents

Stent types present in this cohort included Cordis Precise® (four patients), Abbott AccuLink™ (three patients), Abbott Xact™ (11 patients), Gore Viabahn® (three patients), Boston Scientific Wallstent™ (three patients), SilkRoad Enroute® (seven patients), and one undocumented stent performed at an outside hospital. There were no significant differences between stent types in CTA-measured percent stenosis (MVD, p=0.75; MvM, p=0.69; PAR, p=0.81) nor DUS velocity parameters (PSV, p=0.12; EDV, p=0.07; ICA/CCA ratio, p=0.06) between stent types.

Comparison of measurement techniques

**Figure 2:**
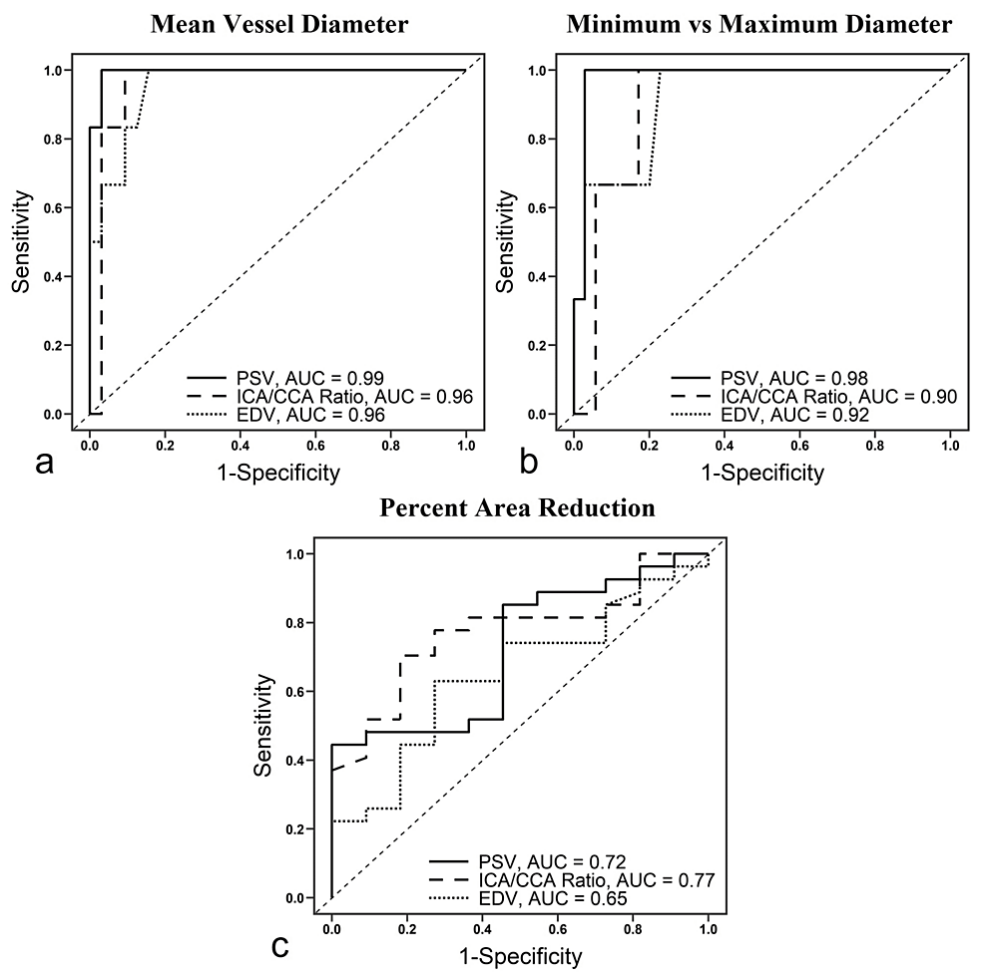
Receiver operating characteristic curves for three measures of percent stenosis on centerline CTA Receiver operating characteristic curves showing peak systolic velocity (PSV, solid line), internal carotid artery and common carotid artery (ICA/CCA) ratio (dashed line), and end-diastolic velocity (EDV, dotted line) area under the curve (AUC) for three different techniques to measure in-stent restenosis. (a) Mean vessel diameter: PSV AUC, 0.99; ICA/CCA ratio AUC, 0.96; EDV AUC, 0.96 (b) Minimum vs maximum diameter: PSV AUC, 0.98; ICA/CCA ratio AUC, 0.90; EDV AUC, 0.92 (c) Percent area reduction: PSV AUC, 0.72; ICA/CCA ratio AUC, 0.77; EDV AUC, 0.65

Excellent interobserver reliability for CTA measurements of percent stenosis was observed for all methods (MVD, ICC=0.95; MvM, ICC=0.94; PAR, ICC=0.94). The three CTA measures of percent stenosis and three DUS parameters i.e., PSV, EDV, and ICA/CCA ratio, were compared using the AUROC curves. All ROC curves can be found in Figure 2 and a comparison of absolute AUC values is detailed in Table 2. Notably, when comparing PSV, EDV, and ICA/CCA ratios, we found no statistically significant difference in their ability to predict a ≥60% ISR across all CTA measurement techniques. Furthermore, we found the MVD and MvM methods to both be accurate CTA measuring techniques based on AUC, but the MVD model was constructed with more paired studies measuring ≥60% ISR compared to the MvM model (six vs three, respectively). Thus, further analysis was conducted using MVD measurements for percent stenosis.

**Table 2:** Comparison of area under the receiver operating characteristic curve for different CTA in-stent restenosis measurement techniques and DUS velocity parameters *Corrected for multiple comparisons by false discovery rate control PSV: Peak systolic velocity, EDV: End-diastolic velocity, ICA: Internal carotid artery, CCA: Common carotid artery, AUROC: Area under the receiver operating characteristics, DUS: Duplex ultrasound, CTA: Computed tomographic angiography

	AUROC for CTA Measurement Techniques			P-values for Comparison of DUS Parameters		
Measurement Technique	PSV	EDV	ICA/CCA Ratio	PSV vs EDV	PSV vs ICA/CCA Ratio	EDV Vs ICA/CCA Ratio
Mean Vessel Diameter Reduction	0.99	0.96	0.96	0.43	0.43	0.96
Minimum vs Maximum Diameter	0.98	0.92	0.90	0.62	0.62	0.88
Difference in Area	0.72	0.65	0.77	0.66	0.66	0.66

Threshold determination

Six (16%) paired studies measured ≥60% ISR and 32 (84%) studies measured <60% ISR by MVD. The mean PSVs, EDVs, and ICA/CCA ratios for ≥60% and <60% ISR cohorts are listed in Table 3 and were significantly different for all DUS velocity parameters (p<0.001). Sensitivity, specificity, PPV, and NPV were then calculated for a variety of PSV, EDV, and ICA/CCA ratio thresholds and are shown in Table 4. The optimal cutoffs determined by maximizing Youden’s index were a PSV ≥240 cm/s (Youden’s, 97%; sensitivity, 100%; specificity, 97%), an EDV ≥50 cm/s (Youden’s, 84%; sensitivity, 100%; specificity, 84%), or an ICA/CCA ratio ≥2.2 (Youden’s, 91%; sensitivity, 100%; specificity, 91%). Maximum specificity was associated with a PSV ≥300 cm/s (100% specificity), an EDV ≥90 cm/s (100% specificity), or an ICA/CCA ratio ≥2.5 (97% specificity). No combination of PSV, EDV, and/or ICA/CCA ratio thresholds was assessed in this study.

**Table 3:** Comparison of mean DUS velocity parameters between ≥60% and <60% in-stent restenosis cohorts ISR: In-stent restenosis, ICA: Internal carotid artery, CCA: Common carotid artery

	Mean	Range	P-value
Peak Systolic Velocity (cm/s)			
≥60% ISR	342	240-424	<0.001
<60% ISR	118	35-266	
End-Diastolic Velocity (cm/s)			
≥60% ISR	90	53-162	<0.001
<60% ISR	32	10-88	
ICA/CCA Ratio			
≥60% ISR	5.1	2.2-9.3	<0.001
<60% ISR	1.7	0.4-9.5	

**Table 4:** Key statistical parameters informing velocity criteria for ≥60% in-stent restenosis PSV: Peak systolic velocity, EDV: End-diastolic velocity, ICA: Internal carotid artery, CCA: Common carotid artery, PPV: Positive predictive value, NPV: Negative predictive value

	Sensitivity %	Specificity %	PPV %	NPV %	Youden’s Index %
PSV Cutoff (cm/s)					
≥240	100	97	86	100	97
≥300	83	100	100	97	83
EDV Cutoff (cm/s)					
≥ 50	100	84	56	100	84
≥ 90	50	100	100	91	50
ICA/CCA Ratio					
2.2	100	91	67	100	91
2.5	83	97	83	97	80

## Discussion

In-stent velocities and redefined criteria

Accurate velocity criteria to predict internal carotid artery ISR is needed to inform decisions for possible reintervention. Utilizing CTA, we calculated statistical parameters for a variety of DUS-measured values and determined that the optimal threshold for detecting ≥60% ISR is a PSV ≥240 cm/s (Youden’s, 97%; sensitivity, 100%; specificity, 97%). Secondary measures were an EDV ≥50 cm/s (Youden’s, 84%; sensitivity, 100%; specificity, 84%), or ICA/CCA Ratio ≥2.2 (Youden’s, 91%; sensitivity, 100%; specificity, 91%). We found that, for given percent stenosis, peak systolic velocities within stented carotid arteries were higher compared to native carotid arteries, which agrees with prior studies. Thus, current DUS criteria based on native carotid arteries [25] may overestimate restenosis when applying these criteria to stented carotid arteries [12-14]. Some have suggested that velocity within stents is increased due to decreased vessel compliance and stiffening of the vessel secondary to unremoved atherosclerotic plaque [12,26]. This phenomenon is not unique to the carotid arteries and has been observed in other vascular territories, such as the mesenteric and renal arteries [26,27].

Previous work [15,20] has been done to refine DUS velocity criteria for evaluating restenosis in the stented carotid artery using statistical methods similar to this study and the results are summarized in Table 5. However, these studies utilized to varying degrees traditional catheter-based angiography, which can be limited by single-plane projection. In contrast, we utilized CTA alone, albeit with advanced vessel analysis. Furthermore, these prior studies introduced verification bias as the reference measurement (i.e., traditional angiogram or CTA) was only performed on patients with suspected ISR based on elevated DUS velocity. Thus, patients with significant ISR but low DUS velocity parameters would have been missed, resulting in elevated cutoffs. Bosch et al. [21] attempted to address this issue by using pre-scheduled CTA/DUS, but they were unable to conclude severe stenosis due to its low prevalence.

**Table 5:** Comparison of previous efforts to redefine DUS velocity criteria in the stented carotid artery. ISR: In-stent restenosis, PSV: Peak systolic velocity, EDV: End-diastolic velocity, ICA: Internal carotid artery, CCA: Common carotid artery, AUROC: Area under the receiver operating characteristics, DUS: Duplex ultrasound

Study	Lal et al. [20]	AbuRahma et al. [15]
ISR Threshold (%)	≥50%	≥50%
Imaging Pairs	310^*^	215^*^
Pairs above threshold	44 (14%)	19 (8%)
Proposed DUS Criteria		
PSV (cm/s)	≥220	≥224
AUROC	0.99	0.95
Sensitivity (%)	100	99
Specificity (%)	96	90
EDV (cm/s)	N/A	≥88
AUROC	N/A	0.82
Sensitivity (%)	N/A	96
Specificity (%)	N/A	100
ICA/CCA Ratio	≥2.7	≥3.4
AUROC	0.99	0.88
Sensitivity (%)	98	96
Specificity (%)	96	100

In our present study, we reduced verification bias by reviewing all patients who underwent internal carotid artery stenting and had CTA performed within 30 days of DUS for any indication. Out of 38 paired studies, only eight CTAs were performed due to increased velocities within the stent, while the majority were indicated for disease outside of the stented artery. This may explain the EDV and ICA/CCA ratio cutoff values (50 cm/s and 2.2, respectively) we found for ≥60% ISR, which are lower than previously reported and are consistent with criteria for unstented carotid arteries [25,28]. Moreover, the EDV cutoff may be explained by the purported decrease in vessel compliance due to stent placement [12,26]: decreased compliance marginally increases diastolic blood pressure but systolic pressures are more affected, which manifests as a widened pulse pressure. Thus, in theory, stent placement would minimally affect diastolic pressures and therefore EDV, as velocity is proportional to the change in pressure along an artery. However, PSV would still be expected to increase, as above. Conversely, the ICA/CCA cutoff ratio may be explained by altered flow parameters in the CCA after stent placement, resulting in an ICA/CCA relationship similar to native arteries.

Clinical implications

As our EDV and ICA/CCA ratio cutoffs are similar to native arteries, to diagnose ≥60% ISR of the carotid artery we propose a new threshold for PSV of 240 cm/s only. This maximizes sensitivity (100%), but a PSV ≥300 cm/s (100% specificity) may obviate the need for further diagnostic imaging, reducing cost, radiation exposure, and contrast use. We chose a ≥60% ISR threshold to balance sensitivity and specificity, but the significance of any ISR threshold is uncertain as current guidelines for reintervention lack sufficient evidence [29]. Despite this, the European Society for Vascular Surgery recommends reintervention for symptomatic patients with 50% to 99% restenosis within 14 days of symptom onset [30]. Conversely, 97% of late, ipsilateral strokes after carotid stenting occur in patients without significant stenosis, thus medical management has been recommended by some for severe stenosis [29,30]. While the threshold for diagnosis of ISR and its optimal treatment remains debated within the field, the degree of restenosis will likely influence future recommendations.

Limitations

There are limitations to this study that may impact its application The paired imaging cohort studied (n=32) was composed entirely of non-Hispanic whites and 81% were men. Furthermore, there were some demographic differences between the paired imaging cohort and the total population that underwent CAS (n=406). While this likely impacts external validity, it remains unclear exactly what effects sex, race, ethnicity, or comorbid conditions have on velocities through stented carotid arteries and whether threshold recommendations would differ based on these factors. Also, the study was conducted as a retrospective chart review with a performance of surveillance carotid DUS and/or carotid CTA non-standardized among several attending vascular surgeons over the study period. There may still be a selection bias in our sample as patients who underwent carotid CTA for any indication may represent a population with higher rates of ISR. Despite high interobserver reliability measures (i.e., ICC), interobserver bias was likely present during the segmentation of the flow-lumen from the stent wall due to windowing variability. Perhaps the most significant limitations are the low prevalence of ISR observed and the overall small sample size. This is not unique to this study and has led to similar limitations in other works [16,21]. However, the widely referenced works of AbuRahma et al. and Lal et al. achieved larger sample sizes through several methods. Notably, this includes adding large numbers of completion arteriograms. As a vessel undergoes remodeling after stent placement, early imaging may not reflect the alterations in flow dynamics proposed to underline the increased velocities seen within stents in the absence of stenosis, which is the crux of redefining DUS velocity criteria. Thus, measurements from completion arteriograms and DUS increase sample size but may not be valid for long-term surveillance criteria.

Due to the inherent variation in DUS measurement, and the small sample size present in this study, validation of these criteria should be conducted for each vascular lab.

## Conclusions

Velocity criteria to predict internal carotid artery ISR are needed to inform decisions for possible reintervention. Using CTA, we found that a PSV ≥240 cm/s on carotid DUS can predict ≥60% ISR with high sensitivity and specificity. This value can be used as an alternative to current velocity criteria based on native carotid arteries. However, the optimal thresholds for EDV and ICA/CCA ratio were similar to native carotid arteries.
